# Spatial Pattern of Standing Timber Value across the Brazilian Amazon

**DOI:** 10.1371/journal.pone.0036099

**Published:** 2012-05-08

**Authors:** Sadia E. Ahmed, Robert M. Ewers

**Affiliations:** Department of Life Sciences, Imperial College London, Silwood Park Campus, Ascot, Berkshire, United Kingdom; Centre National de la Recherche Scientifique, France

## Abstract

The Amazon is a globally important system, providing a host of ecosystem services from climate regulation to food sources. It is also home to a quarter of all global diversity. Large swathes of forest are removed each year, and many models have attempted to predict the spatial patterns of this forest loss. The spatial patterns of deforestation are determined largely by the patterns of roads that open access to frontier areas and expansion of the road network in the Amazon is largely determined by profit seeking logging activities. Here we present predictions for the spatial distribution of standing value of timber across the Amazon. We show that the patterns of timber value reflect large-scale ecological gradients, determining the spatial distribution of functional traits of trees which are, in turn, correlated with timber values. We expect that understanding the spatial patterns of timber value across the Amazon will aid predictions of logging movements and thus predictions of potential future road developments. These predictions in turn will be of great use in estimating the spatial patterns of deforestation in this globally important biome.

## Introduction

The Amazon is the largest remaining area of tropical forest [1], containing half of the world’s tropical forest biome [2], covering an area of nearly 5 million km^2^ [3] and accounting for approximately 10% of the Earth’s terrestrial net primary productivity and biomass [4]–[5]. It is a highly biodiverse system [6] housing a quarter of all global biodiversity [2]. However it is also a system under threat, with an average of 19,500 km^2^ of forest cleared each year between 1996 and 2005 [7]. The spatial patterns of deforestation are determined largely by the patterns of roads that open access to frontier areas, leaving them susceptible to colonisation and further development [8]–[13]. Kirby *et al*. [14] showed that distance from roads is in fact the strongest predictor of deforestation in the Amazon, and Southworth *et al.* [15] reported that deforestation patterns often closely mirror the pattern of the road network with deforestation rates falling with distance from main roads.

The expansion of the secondary road network in the Amazon is primarily driven by the logging sector [16] and logging is a huge industry in the Amazon, with an estimated US$ 2.5 billion of timber extracted each year [16]. Estimates suggest that the amount of forest that is clear cut each year is matched with an equal area being selectively logged each year; approximately 10,000–15,000 km^2^/year [17]–[18]. Even selectively logged forests can lose more than 40% canopy cover through damage to surrounding trees during extraction and increased fire risks [17]. Additionally, roads used by loggers to access timber also serve to open up frontier areas to colonists who further degrade and deforest [11]–[13].

A key goal of the logging sector, indeed any economically driven sector, is to maximise profits. This forms the basis of many land cover/land use change models, which assume a desire to maximise profit and use profit maximisation to determine potential land uses (e.g. [19]). There are two key aspects to profit; revenue and costs. In the logging industry the amount and value of timber extracted determines revenue. To accurately model deforestation driven by logging, it is, then, important to know the spatial distribution of timber values. The location of valuable timber is important because extraction is usually selective [20]–[21] and loggers ideally want to harvest areas that yield the highest profits, i.e. are situated on high density, high value timber and that are accessible with the least cost.

Knowing the economic value of forests is important for conservation as well. For example, Verissimo *et al.* [20] suggested locations for sustainable ‘Flona’ (national forests that allow sustainable logging) by combining data on protected areas, human occupation and forest value. They identified locations that would be economically viable to harvest but that would also provide biodiversity protection. The suggested a set of locations that covered 34% of Amazonian forest, of which 38% was also of high conservation priority. Further, the Amazon offers a host of ecosystem services, from climate regulation, water regulation and carbon storage, to forest products [1], [22]–[27]. In order to calculate a true cost-benefit analysis of logging in these forests, the value of the ecosystem services provided by the standing forest needs to be quantified and compared with the timber values obtained by felling.

There are various estimates of the timber value in the Amazon; some specific e.g. $15.4 billion [28], and some less specific e.g. ‘several trillion dollars’ [29]. However, timber value across the Amazon is difficult to predict because extensive surveys are labour and cost intensive, thus timber value estimates are often based on modelling. Often when looking at forest value, timber values are estimated in terms of net value (profit). For example, Stone [30] modelled the net value of timber using three price classes (valuable ∼>$300/m^3^, medium ∼200–300, and low value ∼100–175 US$/m^3^) as a decaying function of greater distances from sawmills reducing net value, with the assumption that loggers will extract valuable timber from further away. This approach was also used by Verissimo *et al.* [20] and Merry *et al.* [28] who built on the work of Stone [30], making their spatial models more detailed in terms of industry behaviour.

Although spatial profitability has been modelled across the Amazon, it is interesting to know how much the forest is worth in terms of standing timber value. Therefore we have modelled timber value across the Amazon such that valuable tree stands are shown as valuable irrespective of extraction costs. We used ordinary kriging to generate a value map from RADAMBRAZIL survey data for 11 tree genera that are economically important. Kriging methods have been used before in the Amazon region to estimate the spatial distribution of tree diversity [31], tree species distribution [32] and timber density [33]. Our approach extends that of Arima *et al.* [33] who used kriging to estimate total timber density as a way of determining locations for logging road destinations. Here, we combine data on timber density and value to generate a map of potential timber revenue for the Amazon.

## Analysis

The RADAMBRAZIL [34] survey is a selection of surveys carried out between 1968 and 1978 which aimed to map the natural resources of Brazil. Among the data collected on soils, geology and potential land uses, an extensive survey of vegetation was also carried out. The RADAMBRAZIL data set contains information on a total of 89 families and 513 genera of trees, recording the timber volume of individual trees within plots of known location. We aggregated species data by genus for 2465 RADAMBRAZIL forest plots across the Brazilian Amazon. Timber properties, and thus value, are inevitably heterogeneous within and between groupings; genera within families, species within genera and individuals within species will show variation. For example, within the *Tabebuia* genus, which is generally known for desirable hardwood timber such as *T. guayacan*, one may also find medium weight wood species such as *T. roseo-alba* or even light weight wood species such as *T. cassinoides* [35]. However, it was felt that genus was an appropriate level at which to carry out analyses relating to timber values because 71% [36] to 74% [37] of variation in wood density measures among species is explained by genus affiliation, whereas only 25% to 34% is explained by family affiliation.

A list of the top 15 Amazonian tree genera harvested in terms of volume exported (in 1000 m^3^) and the average price per cubic metre, in US$, was obtained from ITTO (International Tropical Timber Organisation [38]). The average value for each genus (US$/m^3^) was obtained by averaging the total export value (US$/m^3^) over the two years for which data were available (2006 and 2007). We were able to obtain data from both RADAMBRAZIL and ITTO for a total of 11 genera (*Dicorynia, Goupia, Hymenaea, Hymenolobium, Manilkara, Mora, Nectandra, Peltogyne, Swartzia, Swietenia, Tabebuia*), which were used in all subsequent analyses. We multiplied the average export value by the total volume of timber recorded at each RADAMBRAZIL location to obtain a timber value for that genus in that plot. RADAMBRAZIL forest plots were 1 ha in area, so our resulting timber values are in units of US$.ha^−1^. Genus value estimates were summed across all 11 genera in each plot to obtain a total timber value (US$.ha^−1^). Total value data was log-transformed to make it normally distributed (all analyses were done with log-transformed total value data, unless otherwise stated). Timber value data from ITTO was for processed sawn wood, whereas the RADAMBRAZIL timber volume data was for standing timber. Timber volume is lost at many stages of the logging process, meaning that 1 m^3^ of timber represents a different ‘volume’ in the ITTO and RADAMBRAZIL datasets. Measurements of timber volume loss in sawmill operations based in the Eastern Amazon state of Para suggest a conversion efficiency of 35% from raw timber to sawn wood [39]. To account for this loss of timber volume and to standardise ‘volume’ measurements among the two datasets, we multiplied all values derived from RADAMBRAZIL data by a scaling factor of 0.35.

**Figure 1:**
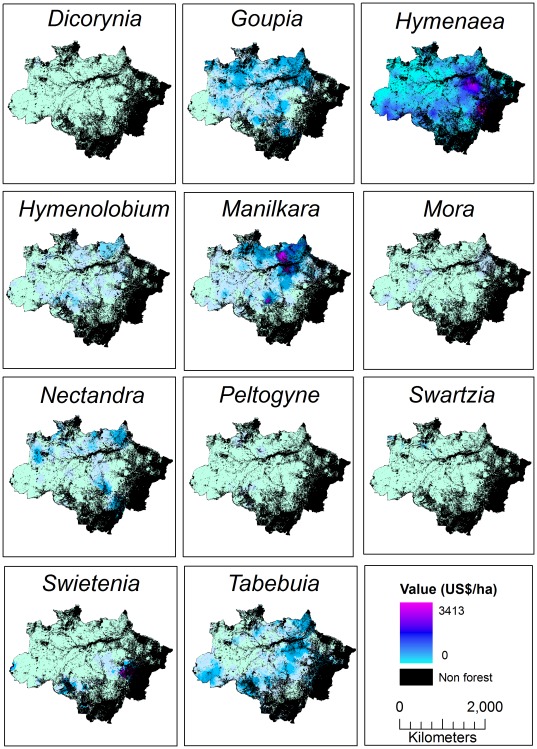
Predicted timber value across the Amazon by genus. Each of the 11 high-value genera, show genus-specific spatial patterns in the distribution of timber value and differences in total value (US$.ha^−1^). *Hymenaea* appears to consistently have the highest value across the Amazon. Other genera, such as *Manilkara, Nectandra* and *Tabebuia* show ‘hotspots’ of high values in relatively restricted areas of the Amazon. Some genera, such as *Dicorynia* and *Peltogyne*, show lower values that do not vary much across space.

**Figure 2:**
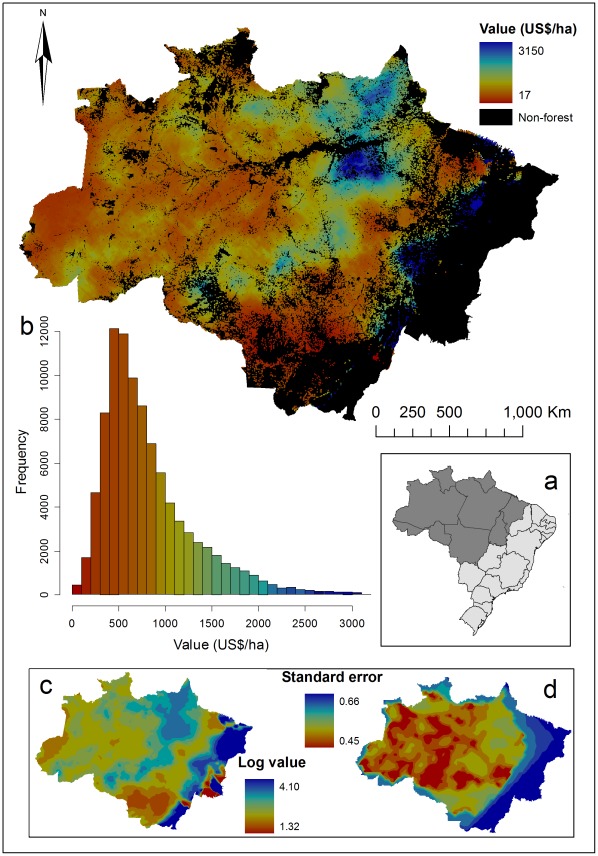
Map of timber value in the Amazon. Values range from low (US$17 per ha) to high (US$3150 per ha). Insets: (a) dark shading shows the spatial extent of the Brazilian Amazon within Brazil, including the state boundaries; (b) frequency distribution of timber values (US$.ha^−1^) in the Brazilian Amazon, calculated over 151,073,784 equal-area grid squares of area 0.25 km^2^; (c) log- transformed timber value across the Amazon without non-forest areas removed (the raw outputs from the kriging analysis). As non-forest areas are not removed the maximum predicted value in panel (c) is 4.10, equating to US$4400 per ha, is higher than the maximum value of US$3150 per ha in panel (a). This discrepancy arises because the maximum values in panel (c) occur along the eastern margins of the Amazon where, in fact, there is little forest standing. (d) standard error of predicted log-transformed timber value.

Ordinary kriging was carried out in ArcGIS 9.3 using the Geostatistical package suite of tools. We initially modelled each of the 11 genera separately (Figure 1), before combining values from all genera within plots (Figure 2) to interpolate total value across the Brazilian Amazon. Output values were anti-logged to give true US$/ha values of timber, after which a forest mask was overlaid on the output map to remove extraneous timber values from areas known to be non-forest and/or previously deforested. We also calculated and present the spatial pattern of error in the kriged values. Because our model was conducted on log-transformed data, we present log-transformed timber values and the standard error associated with that value (Figure 2c,d). An assumption of kriging analysis is that values in locations that are closer in space are more similar than those that are further away. To test for this spatial autocorrelation, we calculated Moran’s I in R 2.10.1 (R Development Core Team [40]) using the ‘pgirmess’ library [41] for total timber value across the Amazon. Moran’s I statistics showed that locations up to ∼200 km were strongly positively correlated (Moran’s I = 0.26–0.19, P<0.001), after which the correlation coefficient drops to <0.04 and is generally non-significant. Furthermore, we validated the un-scaled kriging predictions by using linear regression to model predicted against observed timber value. There was a significant, positive relationship (r^2^ = 0.31, p<0.001, df = 2463) and the regression line did not differ significantly from a 1∶1 relationship (slope = 1.02, 95% confidence interval 0.95–1.09), indicating that the predictions of timber value by kriging were robust.

The 11 genera contributed very different amounts to the overall timber value, and exhibited genus-specific spatial patterns of timber value (Figure 1 & Table 1). The genus *Hymenaea* contributed the most to the total values (mean value US$394 per ha; 95% confidence interval 376–2136), whereas *Dicorynia* contributed the least (mean value US$5 per ha 95% confidence interval 5–10). The mean total timber revenue predicted was US$813 per hectare (95% confidence interval 477–4161), with a distribution showing a few locations with particularly high values of >US$1500 (Figure 2, inset a). There was, however, one area known to be of relatively low value along the eastern edge of the study area that was predicted to be high value. This was likely a result of the kriging interpolation, as there were no survey locations in this area to moderate the predictions. Standard errors on the kriging interpolation (Figure 2, inset d) reflect this, showing a band of high error along the eastern edge of the study area.

**Table 1:** 

Genus	Mean value (US$.ha^−1^)	Lower 95% CI(US$.ha^−1^)	Upper 95% CI(US$.ha^−1^)	Number oflocations	Value(US$.m^−3^)
*Dicorynia*	5	5	10	21	158
*Goupia*	221	221	587	824	257
*Hymenaea*	394	376	2136	945	569
*Hymenolobium*	18	18	26	261	96
*Manilkara*	208	208	1438	598	281
*Mora*	14	14	27	98	216
*Nectandra*	179	173	523	1100	292
*Peltogyne*	20	20	34	255	143
*Swartzia*	42	42	57	909	105
*Swietenia*	40	40	250	57	1096
*Tabebuia*	107	107	232	757	317
Total	813	477	4161	2465	

Mean predicted value of timber (to the nearest US$.ha^−1^, plus 95% confidence intervals) across the Brazilian Amazon by genus, as calculated over 151,073,784 equal-area grid squares of 0.25 km^2^ (data presented in Figures 1 and 2). In some cases the lower 95% CI is the same as the mean, reflecting the heavily left-skewed kriging predictions (i.e. Figure 1b). The number of RADAMBRAZIL locations each genus was recorded at and the average export value (US$.m^−3^) are also shown.

## Discussion

The predicted timber revenue values are comparable to other estimates of timber value in the Amazon. For example, a report by Nepstad *et al.* [42] predicts a maximum *net* value of timber in the Amazon to be US$550, whereas we predict a *gross* maximum value of US$3150 and mean of US$813 per hectare. Once the costs of converting standing timber into sawn wood are taken into account, our figures are comparable. However spatial patterns of high value timber differ between our predictions and those of Nepstad *et al.* [42], primarily because they considered net value (i.e. profit) while we consider gross value. So, while we find the highest value areas to be concentrated in the north east, they reported that high value areas were concentrated around transport systems that offer cost effective access to the forest.

The differences among genera in their predicted values can be attributed to several factors, of which the first is variation in the spatial distributions of the genera themselves. For example, *Dicorynia* is mainly recorded in the north-west of the Brazilian Amazon and was only present in 21 of 2465 locations. Other genera, for instance *Hymenaea, Nectandra* and *Swartzia we*re well represented, being present in 945, 1100 and 909 of 2465 locations respectively. They are also relatively evenly distributed across the Brazilian Amazon rather than being clustered in a small region, which influences kriging predictions. Second the value per cubic metre also varies considerably between genera, for example the average value of *Swartzia* species is US$105 per ha whereas the average value of *Tabebuia* is US$317 per ha, further influencing the different predicted values of the 11 genera. Third, differences in the abundance (i.e. volume) of each genus influenced the predicted value, for instance *Dicorynia* had a total recorded volume of just 101.24 m^3^ whereas *Hymenaea* had a total recorded volume of 4528.30 m^3^. As a result of these three differences among genera, when their individual values are combined the genus-specific patterns are masked and the total values across the Amazon average out. Thus, simply adding the 11 separate genus level kriging predictions does not produce the same results as kriging the total value directly. This averaging effect explains why the maximum genus-specific value obtained of US$3143 per ha is slightly higher than the maximum total value which is US$3150 per ha.

There is a clear spatial pattern in timber value across the Amazon (Figure 1), with the most valuable timber in the north eastern region. Various studies have established that there is an east to west gradient in average wood density/wood specific gravity, with high wood densities occurring in the east [36]–[37], [43]–[44]. This pattern in wood density is also associated with gradients of increasing seed mass in the east [43], higher above ground live biomass (AGLB) in the northeast and central Amazon [45], and a threefold variation in coarse wood production with higher production in the west [44], [46].

The northeast region of the Amazon has old, nutrient poor, well drained soils and a moderately seasonal climate that is occasionally subject to drought [47]. By contrast, the less valuable areas sit in the west and to the south, where there are richer soils, a more seasonal climate and a more dynamic environment in terms of individual tree turnover [43], [47]. These edaphic (soil) and climatic conditions help to explain the emergent pattern of timber value we have presented. ter Steege *et al*. [43] identified two primary gradients in tree composition in the Amazon; the first gradient parallels a major gradient in soil fertility, and the second composition gradient is related to climate, specifically dry season length. They found that in the east the most abundant genera are legumes, yet none of the most abundant genera in the western Amazon are legumes. Thus, unsurprisingly, the poor soils of the east appear then to favour species that are able to cope with low nutrient levels, these species tend to be long-lived trees with slow growth rates but high wood density. Conversely, the more fertile soils in the west are associated with higher growth rates [46], lower wood densities [36], high productivity and a high turnover of individuals [43], [48], with an average stem turnover rate of 2.6%.yr^−1^ in the west/south-west compared to a rate of just 1.35%.yr^−1^ in the northeast [49]–[50]. Additionally, ENSO (El-Nino Southern Oscillation) causes episodic droughts in the eastern and central Amazon [51], however it has little affect on rainfall in the south-west. High wood density species often have a lower vulnerability to drought stress [37] and are thus less affected by episodic drought than their faster growing light wood counterparts. Mean stand level wood specific gravity was found to be 15.8% higher in eastern and central Amazonia compared to western Amazonia [36]. Given that high value trees often have a high wood density and take a long time to grow (mean wood density is inversely correlated with wood productivity [52]), the highly productive, high turnover areas of the west are not ideal for slow growth trees. However, the poor soils of the east that competitively favour legumes (family, *Fabaceae*), which includes seven of the 11 valuable genera considered in this study (*Swietenia, Mora, Swartzia, Peltogyne, Hymenolobium, Hymenaea, Dicorynia),* are more suited to slow growing, stress resistant species that are out competed on richer soils.

We were not able to include all economically valuable timber genera in this study, omitting genera such as *Carapa* that have high average prices. It was necessary to select genera which were present both in the RADAMBRAZIL data set and for which export value (US.$m^−3^) data was available. It is reasonable to assume that inclusion of other economically important timber genera could alter the absolute values emerging from our analyses. However, given the ecological similarities among many economically important timber trees, we feel that the general spatial patterns would remain the same, with the highest value tree stands being located in the northeastern region of the Brazilian Amazon. Another point to note is the possibility that allied genera were confused in the field surveys, with genera such as *Swartzia* and *Bocoa* sometimes misidentified. However, we again feel that the general trends found here would be robust to minor errors in the field data, partly because no single genera contributes more than 19.66% of all individuals analysed.

We have shown that the patterns of standing timber value in the Amazon reflect known, large-scale ecological gradients extending across the Amazon, determining the spatial distribution of functional traits of trees which are, in turn, correlated with timber values. We expect that understanding the spatial patterns of timber value across the Amazon will aid predictions of logging movements and thus predictions of potential future road developments. These predictions in turn will be of great use in estimating the spatial patterns of deforestation in this globally important biome.
